# Neurofeedback Therapy for Enhancing Visual Attention: State-of-the-Art and Challenges

**DOI:** 10.3389/fnins.2016.00352

**Published:** 2016-08-03

**Authors:** Mehdi Ordikhani-Seyedlar, Mikhail A. Lebedev, Helge B. D. Sorensen, Sadasivan Puthusserypady

**Affiliations:** ^1^Division of Biomedical Engineering, Department of Electrical Engineering, Technical University of DenmarkLyngby, Denmark; ^2^Department of Neurobiology, Duke UniversityDurham, NC, USA; ^3^Center for Neuroengineering, Duke UniversityDurham, NC, USA

**Keywords:** visual attention, electroencephalography, brain-computer interface, feature extraction

## Abstract

We have witnessed a rapid development of brain-computer interfaces (BCIs) linking the brain to external devices. BCIs can be utilized to treat neurological conditions and even to augment brain functions. BCIs offer a promising treatment for mental disorders, including disorders of attention. Here we review the current state of the art and challenges of attention-based BCIs, with a focus on visual attention. Attention-based BCIs utilize electroencephalograms (EEGs) or other recording techniques to generate neurofeedback, which patients use to improve their attention, a complex cognitive function. Although progress has been made in the studies of neural mechanisms of attention, extraction of attention-related neural signals needed for BCI operations is a difficult problem. To attain good BCI performance, it is important to select the features of neural activity that represent attentional signals. BCI decoding of attention-related activity may be hindered by the presence of different neural signals. Therefore, BCI accuracy can be improved by signal processing algorithms that dissociate signals of interest from irrelevant activities. Notwithstanding recent progress, optimal processing of attentional neural signals remains a fundamental challenge for the development of efficient therapies for disorders of attention.

## Introduction

The visual system in both human and non-human organisms transforms complex input information into robust neural representation of the visual world. Because the amount of information can only decrease during stochastic neural processing, it is crucial for the visual system to selectively process behaviorally relevant information (Sprague et al., 2015). For instance, when a driver approaches a busy intersection it is important to detect and respond to the relevant traffic light rather than any light source in the visual scene. Attention is the ability to block the irrelevant information to the current task and to enhance the processing of the important information. This key neural function can deteriorate due to some disorders. Patients with disorders of attention are unable to allocate their focus of attention continuously to one task or easily get distracted by irrelevant information. One of the most common disorders of attention, attention deficit hyperactivity disorder (ADHD), is a mental condition characterized by inattention, hyperactivity and impulsivity. ADHD symptoms are dominant in childhood, and extend to adulthood in 15–40% of cases (Biederman et al., 2000; Faraone et al., 2006). ADHD impairs performance in academic, occupational and social tasks (Fleming and McMahon, 2012). According to a meta-regression analysis of 102 studies, ADHD has 5% prevalence worldwide (Polanczyk and Rohde, 2007; Skounti et al., 2007; Millichap, 2008). Treatment strategies have been mostly pharmacological, such as prescription of psychostimulants. However, long-term treatment with pharmacological agents is hindered by side-effects (Conners et al., 2001; Greenhill et al., 2001). Children develop anxiety symptoms after being treated with psychostimulants for 6 months and longer (Vance et al., 1999). There is also a considerable risk of drug misuse and abuse (Kollins, 2008; Steiner et al., 2014a). Psychological therapy, an alternative approach, relieves ADHD symptoms in 30% of cases (Zarin et al., 1998). Overall, currently available therapies for ADHD are only partially effective.

**Figure 1:**
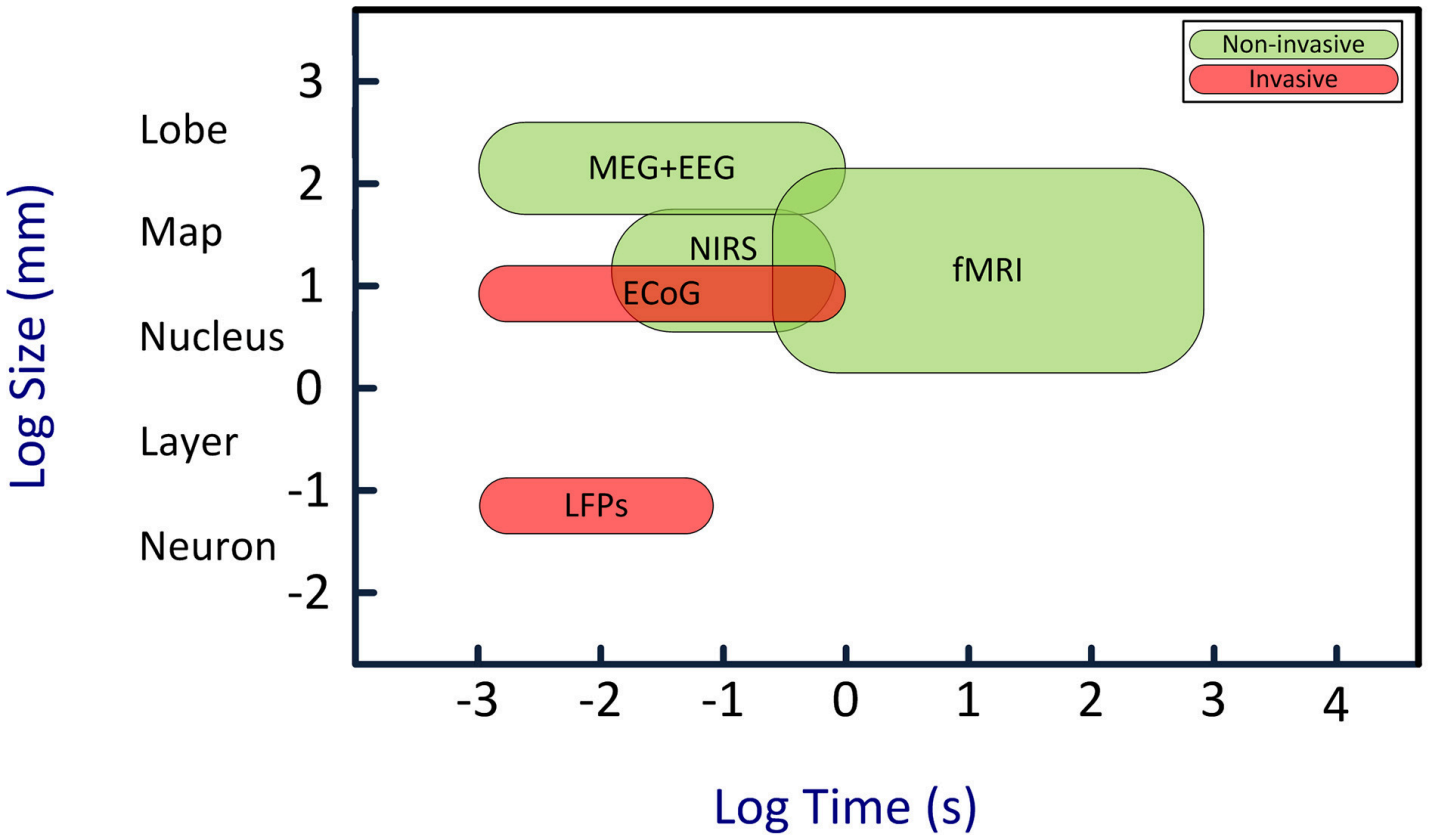
**Temporal and spatial resolution of different BCI techniques**. Although EEG has a relatively poor spatial resolution, its high temporal resolution is an adequate characteristic for real-time BCIs. Abbreviations: EEG, electroencephalography; MEG, magneto-encephalogram; NIRS, near-infrared spectroscopy; fMRI, functional magnetic resonance imaging; ECoG, electro-corticogram; LFPs, local field potentials. Image is inspired from Van Gerven et al. (2009).

Here we review a novel strategy for enhancing the attention capability in patients with disorders of attention. This strategy is based on brain-computer interface (BCI) approach (Arns et al., 2009; Lim et al., 2010, 2012). BCIs establish uni- or bidirectional communication between the brain and external devices (Wolpaw et al., 2000; Donoghue et al., 2004; Lebedev and Nicolelis, 2006; Nicolelis and Lebedev, 2009; Lebedev, 2014; Schwarz et al., 2014). BCIs decode neural signals using mathematical algorithms. Such decoding often utilizes templates of neural patterns defined based on prior knowledge of the characteristics of different neural states. A computer algorithm then compares neural activities with the set of templates to find the best match and determine the current neural state. Additionally, the algorithm can evaluate how well the brain signals match certain requirements, and generate a feedback based on the difference. Such feedback can be used to improve neural function in patients: patients observe their own brain activity in real time, and learn to self-regulate this activity in order to bring it to normal state. This paradigm is called “*neurofeedback*” and the corresponding therapeutic approach is called “*neurofeedback therapy.*” BCIs for humans most commonly utilize electroencephalographic (EEG) recordings (Kus et al., 2013; Tonin et al., 2013; Bamdadian et al., 2014; De Vos et al., 2014; Kashihara, 2014; Yang et al., 2014). Additionally, BCIs can employ magnetoencephalography (MEG) (Mellinger et al., 2007; Bianchi et al., 2010; Ahn et al., 2013), near infrared spectroscopy (NIRS) (Coyle et al., 2004; Sitaram et al., 2007; Power et al., 2012; Waldert et al., 2012; Khan et al., 2014), functional magnetic resonance imaging (fMRI) (Logothetis, 2003; deCharms et al., 2005; Ruiz et al., 2013; Sato et al., 2013), electrocortigraphy (ECoG) (Freeman et al., 2003; Leuthardt et al., 2004, 2009; Schalk, 2010), and multi-electrode intracranial implants (Nicolelis and Ribeiro, 2002; Carmena et al., 2003; Nicolelis et al., 2003; Lebedev et al., 2005, 2011; Zacksenhouse et al., 2007; Peikon et al., 2009; Ifft et al., 2013; see Figure 1 for comparison).

Neurofeedback therapy is applicable to a number of neurological disorders of attention (Lofthouse et al., 2012b; Hillard et al., 2013; Gevensleben et al., 2014; Steiner et al., 2014c; Zandi Mehran et al., 2014). Attention-based neurofeedback paradigms for ADHDs are usually based on visual attention (Arns et al., 2014). As to recording methods, some (EEG, NIRS, ECoG) have been already shown effective for attention control and for treatment of ADHDs, whereas the applicability of others, such as MEG and fMRI, is being researched (Ahn et al., 2013; Sulzer et al., 2013; Sokunbi et al., 2014; Stoeckel et al., 2014; Bruhl, 2015; Okazaki et al., 2015). Implementing an attention-based BCI is a challenging task because the neural representation of attention is highly complex (Ming et al., 2009; Rossini et al., 2012). A good understanding of neurophysiology of attention is required to extract attentional signals from neural recordings and dissociate them from the other ongoing activities in the brain (Sanei and Chambers, 2008). Notwithstanding these difficulties, visual-attention based BCI systems have been already developed and applied to ADHD (Christiansen et al., 2014; Heinrich et al., 2014; Holtmann et al., 2014a,b; Micoulaud-Franchi et al., 2014; Steiner et al., 2014b). In this article, we cover the current state of the art and future challenges in this research.

## Decoding of visual attention from neural signals

### Neural mechanisms of visual attention

In everyday life, we constantly deal with multiple sensory streams from our complex and dynamic environment. The brain starts the processing of this incoming information by filtering out irrelevant signals, which are not consciously experienced because of the filtering. Only a tiny amount of the incoming information enters the higher-order processing levels and becomes available to consciousness (Posner, 1994, 2012). Selective attention is a key function that enables the brain to effectively use its limited information processing capability when confronted with an immense number of inputs from all sensory modalities. High-level cortical areas, particularly the areas of the frontal cortex, play a key role in attentional control. It has been long known that damage to prefrontal cortex (PFC) causes mental deficits which are consistent with a loss of attentional control (Ferrier, 1876). Neurophysiological and functional neuroimaging studies by Posner's group (Fan et al., 2005; Posner and Rothbart, 2007; Petersen and Posner, 2012) have provided a wealth of information on cortical circuitry for attentional control. The main conclusion of these studies is that attention is controlled by a network of interconnected areas that also involved in oculomotor control. These areas include the frontal eye field (FEF), parietal areas and subcortical structures, importantly superior colliculus. This attentional selection network works together with yet another, overlapping network of areas that sustains the focus of attention, called *sustained attention*. The latter system maintains the focus of attention on the selected stimulus. It is composed of the parietal cortex, right frontal cortex and locus coeruleus (Corbetta et al., 2008). Volumetric analysis in ADHD subjects showed that they have smaller frontal cortex compared to healthy subjects (He et al., 2015). This finding explains the deficits in both selective and sustained attention (Pritchard et al., 2008; Avisar and Shalev, 2011; Gomes et al., 2012; Wang et al., 2013). Notably, attention-based BCIs usually require both selecting a visual target and focusing on it (i.e., selective attention) and mental endurance training (i.e., sustained attention).

Selective attention is not a unitary process; it is driven by distinct functional sub-processes associated with different selection criteria (Brosch et al., 2011). Two major sub-processes are: stimulus-driven (exogenous) attention and observer-dependent (endogenous) attention. *Exogenous attention* is driven by intrinsic low-level features of sensory inputs (Egeth and Yantis, 1997; Wolfe and Horowitz, 2004). Low-level properties include such features as stimulus intensity, color and contrast. They all trigger involuntary responses. *Endogenous attention* refers to selection of a target based on an internal state and conscious expectation of a specific object or location (Posner et al., 1980; Desimone and Duncan, 1995). Endogenous selection is performed based on the current aim of the observer. In the classical Posner experiment that dissociated endogenous and exogenous effects, participants were instructed to press a button in response to a visual stimulus that appeared either to the left or right of a central fixation point (Posner, 1980). They were asked to keep their eyes fixed at the center of the screen throughout the task and covertly (i.e., without looking at the target) attend to the peripheral location. To guide this covert attention, a symbolic cue was presented at the center of the screen, which instructed the location to attend. This cue preceded the stimulus onset, and correctly specified the upcoming stimulus location in 80% of the trials. In the remaining 20% of trials, the target appeared at a location that disagreed with the cue. This study showed that the reaction time was significantly shorter when the stimulus was presented at the attended location than when it appeared in the opposite location and there was a misalignment between the endogenous and exogenous attention. Busse et al. investigated neurophysiological mechanisms underlying these two types of attention (Busse et al., 2008). They recorded from single neurons in macaque middle temporal area while monkeys' endogenous and/or exogenous attention was manipulated by the task events. They used a double-cueing paradigm where the first cue instructed the monkey to attend (endogenous attention) to one of three moving random dot patterns (RDPs) until a second cue. The second cue was unpredictable, and therefore captured exogenous attention. It signaled to either shift or maintain the current focus of attention. Findings of this experiment showed that the neural activity was enhanced when attention was endogenously shifted to the first cue. Then, attention was exogenously attracted to the second cue, which was manifested by a transient interruption of neural activity for approximately 70 ms, after which the endogenous attention restored neural representation of the previously relevant stimulus. These results showed that the interruption of endogenous attention by exogenous attention is not a simple refocusing to the new stimulus. Rather, there are separate ongoing processes with distinct neural correlates for endogenous and exogenous effects, as well as an interaction between these mechanisms.

Both endogenous and exogenous attention can be maintained with and without eye movements (i.e., overtly or covertly, respectively). The premotor theory of attention (Rizzolatti et al., 1987) suggests that essentially the same neural circuits in the frontal and parietal areas control the orientation of both overt and covert attention. For overt shifts of attention, eye movements are prepared and executed, whereas for covert shifts they are prepared but not executed. The premotor theory of attention is supported by the fMRI studies showing an overlap between the frontal and parietal regions activated for covert and overt attentional tasks (de Haan et al., 2008). Additionally, neurons in the intermediate layer of superior colliculus which has been long known for their involvement in saccades, are also engaged in the covert attentional shifts (Ignashchenkova et al., 2004). Golla et al. reported a clinical case of impaired overt attention in a cerebellar disorder, and suggested that the cerebellum plays a role in spatial attention (Golla et al., 2005).

Lebedev, Wise and their colleagues compared neural representation of attention with the representation of other behavioral variables, such as spatial memory, target of movement and gaze angle, which often coincide with the orientation of attention, but still can be controlled independently by the brain. In one study (Lebedev and Wise, 2001), they compared neuronal activity in monkey's dorsal premotor cortex (PMd) that reflected the orientation of selective spatial attention with neuronal activities that represented motor preparation, gaze angle, and saccades. The monkeys' attention was attracted by a robot, to which they attended in order to know when to initiate a reaching movement. The target of movement varied. It was either the location of the feeder mounted on the robot or a location of a different feeder. This study showed that approximately 20% of PMd reflected the orientation of selective spatial attention, which could be disengaged from the other spatial variables. These attention-tuned PMd neurons could account for gaze-independent (covert) attentional effects in behaviors with stimulus-response incompatibility. In another study (Lebedev et al., 2004), Lebedev et al. tested the theory that the main function of prefrontal cortex (PF) is the maintenance of working memory. To investigate alternative possibilities, activity of PF neurons was recorded while the monkeys performed an oculomotor task that required remembering one location, but attending to a different location. The largest subpopulation of PF neurons was linked to attention, not to working memory, which indicated that PF has a major contribution to selective spatial attention. Consistent with these findings, studies in human subjects demonstrated the crucial role of frontal cortex in ADHDs (Praamstra et al., 2005; Dirlikov et al., 2015). Dirlikov et al. (2015) used brain imaging technique to explore the cortical morphology in 93 children with ADHD. They found a reduction in cortical surface of PF and premotor cortex (Dirlikov et al., 2015). Several neuroimaging studies suggested that visual attention is controlled by a network of cortical areas interconnected with the FEF. Gray matter is substantially affected in ADHD in the structures of this network, including dorsal and ventral prefrontal cortices, dorsal anterior cingulate area and inferior parietal cortex (Valera et al., 2007; Szuromi et al., 2011). Jonkman and colleagues suggested that the frontal lobe performs early selective filtering, and disorders of this function cause ADHD (Jonkman et al., 2004). A recent resting-state EEG study also suggested that frontal cortex abnormalities are a reliable marker for ADHD (Keune et al., 2015).

Neural oscillations is another neural marker of attention. Oscillations represent synchronous activity of neuronal populations of different sizes, from local to very large. They can be detected in local field potentials (LFPs) recorded with invasive electrodes, or EEGs recorded non-invasively from the scalp (Kahana, 2006). Oscillations are conventionally classified into five frequency bands: δ (1–4 Hz), θ (4–8 Hz), α (8–12 Hz), β (12–30 Hz), and γ (30–80 Hz). Attentional effects have been reported for each of these bands. For instance, attending to a spatial location and anticipating a stimulus at that location is associated with α rhythm attenuation (Rohenkohl and Nobre, 2011). α oscillations are involved in attentional gating of information flow between brain regions (Fu et al., 2001; ter Huurne et al., 2013). To investigate the relationship between brain oscillations and ADHD, ter Huurne used a motion coherence detection task where subjects were instructed to direct attention to either left or right visual field. The attended stimulus was a random dot kinematogram, a field of chaotically moving dots. Subjects were instructed to respond after the dot pattern started to move coherently in the horizontal, but not vertical dimension in the attended hemifield. Dot movements in the unattended hemifield had to be ignored. In healthy subjects, lateralized and sustained α oscillations were detected in the visual cortex during the period when the subjects prepared to respond. In patients with ADHD, oscillations started, but they were not sustained and often stopped before the stimulus onset. Furthermore, lateralization of α oscillation was highly correlated with the degree of spatial attention in the healthy group, but not in the ADHD group (ter Huurne et al., 2013). In neurofeedback training experiments, children with ADHD were able to increase α-power following 18 training sessions (Escolano et al., 2014). Overall, these studies suggest that brain oscillations can be used to monitor neural regulation of attention and improve it using neurofeedback therapy.

### BCIs for visual attention

Early attempts to treat disorders of attention using neurofeedback date back to the mid-eighties and nineties (Elbert et al., 1980; Lutzenberger et al., 1980; Wolpaw et al., 1991). Since then, considerable progress has been made in the development of computer algorithms for the decoding of attention-related neural signals. In a typical setting, subjects endeavor to keep their visual attention while playing a video game. Attention related brain signals are extracted from the neural recordings and fed back to the subjects using visual feedback. Successful performance is rewarded. Repeated training sessions with such a BCI system engage brain plasticity mechanisms, and eventually improve attention (Dobkin, 2007; Rossini et al., 2012).

Both invasive and non-invasive recordings have been used in BCIs. Invasive BCIs utilize electrodes that penetrate the brain (LFPs and single-unit recordings) or are placed on the brain surface (ECoG). These systems require an invasive surgical procedure to implant the electrodes. Non-invasive BCIs, on the other hand, do not require any surgery and can be safely and easily implemented. Non-invasive sensors are placed on the scalp (EEG, fNIRS), or in some implementations make no contact with the head (fMRI, MEG) (see Table 1 for details). Additionally, *hybrid* or *multimodal* BCIs employ combinations of different recording methods in order to improve performance. Fazli et al. (2012) developed a multimodal BCI consisting of the combination of EEG and NIRS that improved the signal classification accuracy in 90% of participants. That multimodal BCI had higher sensitivity and specificity and were resistant to environmental noise. Such combined EEG-NIRS neurofeedback can be used by subjects who cannot operate a BCI solely by their EEG activity (Fazli et al., 2012).

**Table 1:** **Comparison of different signal acquisition methods used for BCI application**.

**BCI method (measured signal type)**	**Advantages**	**Disadvantages**
LFPs (Firing rate of bundles of neurons)	High SNR; low variability during the experiment; targeting the activity in specific brain areas; higher resolution of detecting temporal and spatial features in several parallel-activated brain regions.	Intracranial surgery; very susceptible to signal-loss in long-term implantation (Shain et al., 2003; Donoghue et al., 2004); requires precise source localization in order to implant the electrodes in the right location; less common in human studies.
ECoG (Electrical activity from brain surface)	Supports accurate BCI operation with little training (Leuthardt et al., 2004); higher spatial resolution and amplitude than EEG (Freeman et al., 2000; Leuthardt et al., 2009); far less EMG and EOG artifacts (Freeman et al., 2003; Ball et al., 2009); greater long-term functionality compared to LFPs (Margalit et al., 2003); more stable SNR compared to EEG (Schalk, 2010).	Intracranial surgery; Limited long-term functional stability and signal loss (Schalk and Leuthardt, 2011); very rare research application (Sutter and Tran, 1992).
EEG (Electrical activity from the scalp)	Superior temporal resolution (suitable for real-time experiments); ease of use (non-invasive) even by inexpert individuals; inexpensive (compared to other devices); least ethical concern and medical risks compared to other methods; portable.	Susceptible to noise (EMG, EOG and environmental); Low spatial resolution (harder to localize brain activities); requires a substantial degree of user training in BCI development.
fMRI [Blood oxygenation level dependent (BOLD)]	Superior spatial resolution (deCharms et al., 2005; Lee et al., 2009); signal detection from whole brain including the subcortical structures (Logothetis, 2003; Weiskopf et al., 2004).	Signal drift due to imperfection of magnetic gradient field (Lee et al., 2009); limited to BOLD-signal-based analysis (can be done in ERP experiments but not in frequency-range analysis); less suitable for real-time BCI due to low temporal resolution; strict physical restriction of subjects inside the scanner due to motion artifacts; requires expensive devices and expertise to operate the system.
NIRS (Measure of oxygenated hemoglobin)	Robust when dealing with noise (Coyle et al., 2004; Waldert et al., 2012); superior in detection of stimulation onsets and offsets (reducing the false positive commands) (Tomita et al., 2014); precise parameter setting to extract features is not needed to detect information on the brain (Kanoh et al., 2009).	Lower temporal resolution compared to EEG (Tomita et al., 2014).
MEG (Magnetic field)	Higher spatiotemporal resolution (Mellinger et al., 2007) than EEG; less training sessions than EEG; more robust in detectability of different frequency-band compared to EEG (Mellinger et al., 2007).	Expensive (at least 10 times more expensive than EEG cost) and non-portable; lower spatial resolution compared to fMRI; poorer localization for deeper brain structures compared to fMRI.

The research on BCIs that improve attention has experienced a steady growth, especially BCIs for ADHD patients. Some of these results are controversial. A number of studies reported positive outcome of neurofeedback training (Leins et al., 2007; Gevensleben et al., 2009; Steiner et al., 2011; Wangler et al., 2011), whereas others questioned these findings. In the camp of neurofeedback advocates, Arns et al. (2009) analyzed literature on neurofeedback therapy for ADHD and concluded that this treatment was “efficient and specific” (Arns et al., 2009). Lofthouse et al. (2012a) called this therapy “probably efficacious” based on their analysis of research conducted from 1994 to 2010, where the majority of studies utilized θ/β ratio (see below) (Lofthouse et al., 2012a). However, Vollebregt et al. (2014a) came to a different conclusion in their systematic review of frequency-band based BCIs for ADHD. They concluded that there was no significant effect of treatment on any neurocognitive variables affected by ADHD (Vollebregt et al., 2014a). This negative result highlights the need for further research on EEG features that would better suit attention-based BCIs. Here we review these features and the ways they could be used to improve neurofeedback therapy for ADHD.

## Feature extraction for visual-attention BCIs

Feature extraction is a critical part of BCI implementation and design (Shahid and Prasad, 2011). During this processing stage, a specific characteristics are extracted from brain recordings, which are then decoded and converted into control commands or neurofeedback. Depending on the recording method, different features can be used. For example, single-unit recording are usually converted into neuronal firing rates, and EEGs are converted either into the spectral power or parameters of event related potentials. The selection of features depends on the way the user communicates with the BCI system. In the BCI design called endogenous BCI, subjects self-generate neural patterns (Nicolas-Alonso and Gomez-Gil, 2012). Alternatively, in the BCI design called exogenous BCI, neural responses are evoked by an external stimulus, and subjects modulate these responses, usually by focusing attention on relevant stimuli. Table 2 compares these two BCI types.

**Table 2:** **Comparison of endogenous and exogenous BCIs and their corresponding protocols**.

**Category**	**Protocol**	**Advantages**	**Drawbacks**
Endogenous BCI	- Source of the brain activity - Frequency bands	- Independent of any specific task - Useful for patients with sensory deficits - Perfect for freely moving operations (since subjects are not instructed to stare at specific stimulus)	- Requires several sessions of trainings - Some patients may not be able to communicate with BCI (BCI illiterate) - Low information transfer rate - Low signal-to-noise ratio - Low spatial resolution of EEG-based BCIs (harder for source localization analysis) - Requires many EEG electrodes
Exogenous BCI	- ERP - SSVEP	- Low training session - High information transfer rate (explained in the next section) - Feasible with a few EEG channels - Higher signal-to-noise ratio	- System failure if the subject is not attending to the stimuli - Fatigue in subjects (especially in SSVEP tasks due to constant flickering objects)

### Endogenous BCI

#### Attention-based BCIs that utilize neural oscillations

Spectral analysis of EEGs recorded at different scalp locations is commonly used to extract features for endogenous BCIs. Here, time-dependent changes in the EEG spectra for different electrodes are detected using EEG time-frequency (TF) analysis. For example, TF analysis can detect the occurrence of brain oscillations that result from transient synchronization of neuronal discharges over a millisecond time scale (Sanei and Chambers, 2008). This method can be applied to measure EEG changes associated with attention, such as synchronization of specific EEG bands associated with attention to an object. Attention-related synchronization of neural activity can be detected using a variety of recording methods, including single-unit recordings from brain neurons. Fries et al recorded from individual neurons in cortical area V4 while macaque monkeys attended to behaviorally relevant stimuli and ignored distractors (Fries et al., 2001). The neurons increased their gamma-band synchrony while decreasing low-frequency (<17 Hz) synchrony when the monkeys attended. Several studies showed attention related effects in ECoGs. Rougeul-Buser and Buser recorded ECoGs in freely moving cats and observed that 10–14 Hz oscillation over sensorimotor cortex, called μ-rhythm or “expectancy rhythm,” increased when an animal actively attended to a place where a mouse was expected to appear (Rougeul-Buser and Buser, 1997). The μ-rhythm epochs were often followed by a brief 20 Hz (β-range) ECoG burst. Thorpe et al reported topographical details of these ECoG patterns. Attention was associated with μ-rhythm increases over parietal regions, whereas, β-band activity increased in motor areas (Thorpe et al., 2012). Daitch et al. suggested that these oscillatory patterns serve to increase functional connectivity between the areas that process relevant information while suppressing unwanted cross-talk within the neural network areas that could be caused by irrelevant stimuli (Daitch et al., 2013).

EEG studies have shown that high-frequency oscillations (>30 Hz) are correlated with increased attention (Kaiser and Lutzenberger, 2005; Koelewijn et al., 2013; Musch et al., 2014). Similar results were obtained using microelectrode recordings in freely behaving monkeys (Fries et al., 2001). Attention-related oscillations can have the frequency higher than the typical γ -band range (30–80 Hz) (Crone et al., 2006). Ray et al. (2008) presented human subjects with a sequence of tactile and auditory stimuli separated by pseudo-random time intervals. The tactile stimuli were delivered using a tactile stimulating cylinder, which the subjects gripped with their hands. The auditory stimuli were delivered through a headset. The subjects were instructed to attend to one of the two modalities (auditory or tactile) and respond to the attended stimulus with a button press (Ray et al., 2008). The attended stimuli enhanced high γ activity (80–150 Hz) in the cortical areas that processed the corresponding modality: attention to auditory stimuli activated auditory cortex, and attention to somatosensory stimuli activated somatosensory cortex. Additionally, these high-gamma oscillations occurred in PFC irrespective of the attended modality. This result is consistent with PFC being involved in the global attentional system (Dirlikov et al., 2015; Keune et al., 2015) regardless of the modalities of input information. Another study reported that attention in humans was associated with high frequency oscillations of approximately 350 Hz that occurred in frontal and centro-parietal regions in response to somatosensory stimuli (Ozaki et al., 2006). Several hypotheses have been proposed to explain the role of high-frequency oscillations in attention. One hypothesis states that low-amplitude, ultra-high frequency activity is a background neural noise that enhances neural processing (Benzi et al., 1982). For example, adding a small amount of noise to a neural circuit makes its component fire more synchronous (Ward et al., 2006). Here, the performance is improved due to stochastic resonance (Benzi et al., 1982), where high-frequency noise lowers detection threshold for the relevant stimulus-. The stochastic resonance driven by γ waves can play a role in high cognitive functions (Ward et al., 2006). A similar resonance can be produced by injecting noise to the brain using electrical stimulation (Medina et al., 2012).

A number of BCIs for controlling attention have been developed based on EEG spectral bands. A recent study showed that healthy subjects can quickly learn to self-modulate their γ-oscillation in superior parietal cortex by alternating between the attentive and rest states (Grosse-Wentrup and Scholkopf, 2014). This BCI system correctly decoded brain state in 70.2% of cases. Several of studies on attention-based BCIs employed the ratio of power in specific spectral bands as the signal feature to be classified. This ratio was calculated as β/(α+θ) in many reports (Nagendra et al., 2015). The higher the ratio, the higher is the level of attention. Other studies used θ/β ratio (Clarke et al., 2013; Dupuy et al., 2013; Heinrich et al., 2014; Vollebregt et al., 2014b) that decreased with enhanced attention. These ratios reflect the fact that θ and α rhythms are stronger in drowsiness and the inattentive states; whereas, β rhythm is stronger in attentive states. For example, spectral EEG composition prior to stimulus presentation is indicative of the level of visual attention (Busch et al., 2009). Several characteristics of EEG rhythms can be also used to assess the level of attention. Instantaneous phase of EEG oscillations is one such characteristic (Busch et al., 2009). In the experiments of Busch et al. (2009), subjects were instructed to detect a brief (6 ms) light flash presented either at an attended or unattended location. Hit and miss rates were found to be correlated with the phase of EEG oscillations at the time of stimulus presentation. Additionally, stimuli preceded by strong α activity were less likely to be detected, an observation reported in the previous literature (Ergenoglu et al., 2004; Babiloni et al., 2006; Thut et al., 2006; Hanslmayr et al., 2007). In the other study on the relationship between EEG phase and detection of attended and unattended stimuli, Busch and VanRullen (Busch and VanRullen, 2010) analyzed the relationship between the pre-stimulus EEG pattern and the EEG response to the stimulus. They found that EEG responses were higher when EEG was at a certain phase the just prior to the stimulus onset and the EEG response was the lowest for the opposite EEG phase (Busch and VanRullen, 2010). The periodicity of EEG was 100–150 ms in these experiments. Several studies reported similar results (Makeig et al., 2002; Lakatos et al., 2008; Busch and VanRullen, 2010).

### Exogenous BCI

#### Event-related potential (ERP) paradigms

Event-relate potentials (ERPs) represent a compound response to a stimulus of large neuronal populations. An ERP consists of several voltage deflections that occur on a millisecond time scale. Specific ERP components have been linked to different neural origins (Cohen, 2013), including the components that are associated with attention. ERP is one of the most commonly used protocols for attention studies (Wu et al., 2009; Gherri and Eimer, 2011; Jones et al., 2013; Matheson et al., 2014; Zheng et al., 2014). ERPs recorded in primary sensory areas increase when the corresponding stimulus modality is attended to Harter et al. (1984). Selection of the appropriate ERP components and scalp locations to sample is essential to achieve good performance of an ERP-based BCI. The first ERP-based BCI was designed by Farwell and Donchin (1988). Subjects looked at a 6 × 6 matrix of alphanumeric characters. A single electrode was placed over the Pz (central-parietal) site. Subjects were instructed to attend to a specific character within the matrix while rows and columns periodically flashed. Attended stimuli evoked stronger ERPs and thus could be identified. Averaging over 30 trials was required to improve the signal-to-noise ratio and assure BCI accuracy.

For better design of ERP-based BCIs, it is important to take into consideration the detailed sequence of ERPs components. The first component is the C1-wave which is detected mostly by the posterior midline electrodes in the EEG. The onset of the C1-wave is typically 40–60 ms after the stimulus with the peak at 80–100 ms post-stimulus. C1 is generated in the primary visual cortex (Luck, 2014) and its polarity changes as a function of location of the stimulus in the visual field, i.e., whether the response comes from upper or lower bank of calcarine sulcus. This change in polarity has been identified as a unique feature for C1-wave compared to other components and has been used by many studies as a marker for V1 sources. However, later neuroimaging studies challenged this view. Ales et al. (2010) used fMRI retinotopic mapping to identify the location of V1, V2, and V3 overlaid on the high-resolution structural MRI (Ales et al., 2010). This technique allowed them to acquire a 3D shape of the upper and lower visual field projection in V1 and adjacent areas, V2 and V3. Contrary to previous studies, they found that sources in V1 do not fully conform to the sign reversal. Furthermore, V2 and V3 also showed a polarity change for upper and lower field stimuli. This suggested that the polarity inversion criterion was not a reliable method for source localization. Yet another study challenged this conclusion. Kelly et al. claimed that C1 does initiate from V1 (Kelly et al., 2013). It has been also reported that attention is not important to generate the C1 component (Martinez et al., 1999; Fu et al., 2010). According to Martinez et al., although primary visual cortex is involved in attention, it does not serve as the locus of initial sensory gain control for attended and unattended inputs. Kelly et al. (2008) disagreed with this and proposed that attentional selection occurs at the early visual processing stage reflected by C1 generation in V1 (Kelly et al., 2008). In that study, target brightness and location were adjusted for each participant in order to reduce inter-subjective variability of C1. After this correction, it became clear that C1 was enhanced due to spatial attention, which indicated that this early sensory component was adjusted before the visual information arrived in V1.

The second component is the P1-wave that starts 60–90 ms after the stimulus and peaks at 100–130 ms. It contains an early portion generated from middle occipital gyrus and a late-portion generated more ventrally, from fusiform gyrus (Di Russo et al., 2002). P1 is sensitive to the direction of spatial attention (Hillyard and Anllo-Vento, 1998). Luck and Hillyard (1995) studied attentional modulation of P1 using a stimulus display that consisted of 14 gray items and 2 colored items (Luck and Hillyard, 1995). Subjects were instructed to report presence or absence of specific colored-item (feature detection condition) or the shape of a specific colored-item (conjunction discrimination condition). Just after the onset of the search array, a task-irrelevant stimulus appeared either at the location of relevant or irrelevant items. The irrelevant stimulus evoked larger ERPs for the relevant location compared to irrelevant location. P1-wave was present in that ERP only in the discrimination condition and not in the feature detection condition, indicating that conjunction discrimination recruited additional attentional resources. In the traditional paradigm, where subjects are instructed to pay attention to one direction and ignore the other, Mangun et al. (2001) showed that the P1 magnitude is larger for the attended compared to unattended location. The study of Mangum et al. also showed that P1 response was generated not only by the contralateral hemisphere but also by the ipsilateral one, the observation that was difficult to explain in terms of redistribution of attentional resources between the hemispheres. Klimesch (2011) suggested that these results is due to inhibition effect of the P1 in two different levels. In the task-irrelevant pathways (e.g., ipsilateral hemisphere) inhibition is used to block the information processing, whereas, in the task-relevant pathways it is used to increase the SNR by enabling precisely timed activity in neurons with high level of excitation and suppressing the neurons with low-level of excitation. It seems that the inhibition increased when an attentional demand increases to make the response to the relevant stimulus sharper.

N1-wave contains an early component generated in the frontal (140 ms) and two late components between 150 and 200 ms generated parietal cortex and the lateral occipital cortex, respectively (Clark and Hillyard, 1996; Luck, 2005; Ceballos and Hernandez, 2015). The magnitude of N1-potential is highly influenced by visual spatial attention (Hillyard et al., 1998). N1 is insensitive to the physical properties of the paradigm such as light intensity and the contrast. This point was clarified in the experiment where a 6 × 6 matrix alphanumeric matrix (similar to Farwell and Donchin's paradigm) could be either high-contrast or low-contrast (Shishkin et al., 2009). Although the visual stimuli were designed in a very different contrasts, N1 characteristics between high- and low-contrast tasks remained the same. N1 is an interesting feature from two aspects: first N1 seems to be reproduce robust feature regardless of design on the paradigm which makes it suitable to compare different studies; second, there is no need to make detection paradigm hard to enhance N1 as it works well for clearly visible stimuli, and therefore, N1-based BCIs can be visually comfortable for ADHD subjects. This is important for ADHD subjects as they have higher tendency for fatigue or visual discomfort (Cao et al., 2014; Kooij and Bijlenga, 2014). It has been shown that about two-third of children with ADHD suffer from visual problems such as irritability by light (Kooij and Bijlenga, 2014). If BCIs are intended to be used on a daily basis for training and rehabilitation purposes, the rapid visual fatigue would be a great disadvantage (Sakurada et al., 2015). Therefore, presenting a paradigm with less discomfort effect should enhance the endurance of patients in long-lasting training sessions and consequently increase the chance of successful therapy.

N1 properties are influenced by repetitive training which can be a potential marker for evaluating the effect of neurofeedback therapy. For example, training to play a video game affects N1 (Latham et al., 2013). In that study, checkerboard stimuli appeared for a short time (92 ms) either in the left or right hemifield against a gray background. Subjects were instructed to respond to the flash of checkerboards by pressing a button while EEG was being recorded. Participants were divided into two groups: professional video-game players (VGP) and non-professional VGP. Expert VGPs had significantly shorter N1 latencies compared to inexperienced VGPs, and no other difference in ERP components was found between the groups.

P2-wave, that follows N1, occurs mostly for the anterior and central electrodes. P2 is larger when the stimulus occurs relatively infrequently (*oddball*). From this point of view, the anterior P2 is similar to P3-wave (see below) with the difference that P2 represents simple features (e.g., color) of the stimulus, whereas P3 is related to complex stimulus features (e.g., color and shape). For posterior electrodes, P2 component is often difficult to distinguish from the overlapping N1, N2, and P3 (Luck, 2014). P2 magnitude has been reported to differ between healthy and ADHD individuals (Banaschewski et al., 2003, 2008; Broyd et al., 2005). The P2 component is associated with automatic processing and inhibition of irrelevant information (Barry et al., 2003). Studies have shown that P2 has larger amplitude and different topographical distribution in ADHD (Banaschewski et al., 2003; Broyd et al., 2005; Barry et al., 2009; Ortega et al., 2013). Therefore, P2 amplitude could be used in BCIs as an indicator for improvement scale for ADHDs.

P3 component (also called the P300 since it peaks at 300–500 ms post-stimulus) consists of two sub-components P3a and P3b. The P3b amplitude varies between 5 and 15 μV for the parietal electrodes (Soltani and Knight, 2000). It appears following the occurrence of the oddball stimulus among a sequence of frequently repeating background stimuli. P3a, on the other hand, is distributed more in the fronto-central scalp region and peaks about 60–80 ms prior to P3b for all sensory modalities. An important characteristic of P3a component is its habituation in frontal sites within 5–10 stimulus presentations; i.e., the P3a disappears when the same type of stimulus is repeatedly presented (Lynn and Eysenck, 1966; Sokolov, 1969; Friedman et al., 2001). P3b, in many publications, is simply referred to P3 or P300. It was proposed that P3 is a possible endophenotype for ADHD (Doyle et al., 2005; Szuromi et al., 2011). Patients with ADHD have significantly lower P3 amplitude during the attention task (Szuromi et al., 2011). Szuromi et al. (2011) proposed that the P3 may be used as an ADHD marker that characterizes the deficits in the level of attentional allocation and information processing. P3 magnitude has been reported to represent the effort of attentional allocation, whereas, the latency of P3 indexes the processing speed of stimulus evaluation (Polich, 2007). Yet, P3 should be considered conservatively as a unique indicator for attention deficiency since its characteristics can be affected also by other disorders such as externalizing psychopathology (substance use, conduct disorder and antisocial behavior) (Bertoletti et al., 2014; Burwell et al., 2014).

ERP-based BCIs is one of the early developed methods in the field of BCI (Farwell and Donchin, 1988). ERP-based BCIs have a relatively low information transfer rate (ITR) or bit-rate. Bit-rate in a BCI system is an index of how much information can be communicated between the brain and the computer in the time-unit (van der Waal et al., 2012). In Farwell and Donchin's BCI, the ITR was about 12 bits min^−1^. Zhang et al developed a visual P300-speller BCI which was able to communicate at 20 bits min^−1^. BCI performance is substantially higher for visual ERPs compared to auditory (1.54 bit min^−1^) and tactile (7.8 bit min^−1^) ERPs (Furdea et al., 2009; van der Waal et al., 2012). Combination of ERP with other protocols such as steady-state visual evoked potential (SSVEP) increases the ITR up to 19.05 bit min^−1^ (Panicker et al., 2011).

ERP-based BCIs increase SNR by performing an ensemble averaging across several responses. Only the time-locked activities survive the averaging and irrelevant activities are canceled out. However, averaging is also considered as a drawback of ERP-based BCIs as the system has to obtain two or more ERP events to improve performance. Collecting data in multiple trials slows down the system speed. Thus, choosing this ERP-BCI method is a trade-off between the speed and accuracy of the system. Another limitation of ERP-based BCI is the across-trial variability in ERP amplitude and timing. The amplitude of P3 decreases if inter-trial intervals are short. To keep P3 amplitude in the standard range (10–20 μV) inter-trial interval should be around 8 s (Polich and Bondurant, 1997). This long interval limits BCI performance. In most experimental designs, intervals between oddball stimuli are random, which introduces ERP variability. Variability in the P3-characteristics makes it an unstable feature in attention experiments where the rigidity of ERP depend both on factors such as the designed paradigm and the mental states of the subjects.

#### Signal characteristics in steady-state visual evoked potential (SSVEP) paradigms

Another widely used BCI protocol is the SSVEP (Zhang et al., 2010; Palomares et al., 2012; Lesenfants et al., 2014; Wu and Su, 2014; Reuter et al., 2015). Visual evoked potential (VEP) is the brain responses to a visual stimulus such as light flash or flickering of a checker board at a specific frequency (Punsawad and Wongsawat, 2012). Presentation of a flickering visual object leads to VEPs entrained to the stimulus frequency. SSVEP-based BCIs usually detect this entrained response in the EEG of the visual and parietal cortices. These BCIs achieve high SNR over a few seconds of stimulation (Dmochowski et al., 2015). In a typical SSVEP-based BCI, several objects flicker at different frequencies while the subject attends to one the object. The subject usually looks at the attended object. SSVEP-based BCIs can be easily implemented using graphical interfaces such as video games (Leins et al., 2007; Lim et al., 2010, 2012; Bakhshayesh et al., 2011).

SSVEP-based BCIs have good accuracy and resistance to artifacts. As such, they can be used to build practical assistive systems for disabled users (Muller-Putz and Pfurtscheller, 2008). For example, Bin et al reported a SSVEP-based BCI that attained 95.3% accuracy and the ITR of 58 ± 9.6 bits min^−1^ (Bin et al., 2009). This is a substantially higher ITR compared to other BCI types, such as ERP-based BCIs. Muller and Hillyard (2000) designed a paradigm in which ERPs were embedded within a flicker sequence. They found that the magnitude of SSVEP and that of the N1 and N2 component of ERP varied together (positive correlation), whereas no significant correlation was found with other ERP components (Muller and Hillyard, 2000). SSVEP paradigms usually utilize the flickering frequency greater than 6 Hz. In a recent study (Dreyer and Herrmann, 2015), flickering frequency of up to 100 Hz was used by utilizing a novel technology. High-frequency SSVEPs are of great advantage because subjects do not perceive the flicker and are not annoyed. The flicker is not perceived for stimulus frequencies higher than 40 Hz (Lin et al., 2012). Sakurada et al. (2015) demonstrated that using BCIs with SSVEP frequency above 50–60 Hz enhanced the classification accuracy and decreased visual fatigue (Sakurada et al., 2015). Training time is also improved, particularly in ADHD subjects, as they are less irritated by light flicker (Kooij and Bijlenga, 2014).

SSVEPs can be detected not only in awake subjects, but in anesthetized subjects, as well. Several experiments employed the SSVEP technique in fully or partially anesthetized animals whose eyes were kept open in front of a visual display (Harnois et al., 1984; Xu et al., 2013). The flicker frequency was detected from the occipital electrodes.

Harmonics of the flickering frequency in some cases give a better BCI readout (Muller-Putz and Pfurtscheller, 2008; Allison et al., 2010; Ordikhani-Seyedlar et al., 2014). Müller-Putz and his colleagues reported particularly good results when they used three harmonic peaks (Muller-Putz et al., 2005). In our study (Ordikhani-Seyedlar et al., 2014) that employed a covert attention paradigm, the power of the second harmonic was higher compared to the first harmonic. This result is in agreement with Kim et al. (2011) and others Garcia et al. (2013), Zhang et al. (2015) who also reported that visual spatial attention modulates the second, but not the first harmonic of the SSVEP frequency.

## Prospects for BCIs in research of attention

We are witnessing a rapid development of the BCI field. The number of peer-reviewed articles has been rapidly increasing over the past 20 years. Many of BCIs reported in the literature enable sensorimotor functions (O'Doherty et al., 2011; Ifft et al., 2013; Pais-Vieira et al., 2013; Yoo et al., 2013). While BCIs for cognitive functions are less developed, there has been a growing interest to such systems. In our opinion, the most important future challenges for attention-based BCIs include:

(1) Filtering out noise: Noise can be caused by mechanical and electrical artifacts, and it can be a neural signal that is irrelevant to the function that the BCI enables and/or augments. Noise can be reduced by proper selection of features representing a brain function of interest. Choosing the right features is especially important for therapeutic BCIs because if features are selected incorrectly, unwanted functions could be enhanced instead of the desired alleviation of an individual's disability. For instance, using the α-band to regulate attention has certain caveats. Ideally, the α-band represents suppression of irrelevant information in an attention paradigm. However, if the subject is not attending, such suppression could be confused with the drowsiness state, and the BCI would enhance drowsiness instead of working properly to enhance attention. This problem could be addressed by adding features, such as topographical information about the source of the α-oscillations.
(2) Developing of reliable criteria to quantify BCI training effects: Neurofeedback therapy is usually evaluated using a comparison of specific features before and after the training. However, enhancement in EEG features does not guarantee a behavioral improvement. For example, increase in β-band power is a popular feature indicating high attention level. If the aim is just to increase β-band oscillation, this frequency band might also be increased due to some other brain function unrelated to attention *per se*. For example, the β-band increased when motor movement had to be voluntarily suppressed in macaque monkeys (Zhang et al., 2008). Therefore, we suggest that the evaluation of neurofeedback therapy outcome should include behavioral and psychological tests to that evaluate the target function.
(3) Accounting for intra- and inter-individual variability: Sources of variability include non-stationarity of EEG signals (Vidaurre et al., 2011) as well as non-stationarities induced by the task (Iturrate et al., 2013) and different mental states of different subjects. The BCI algorithms should be able to accommodate individual characteristics of subjects, and to adapt to EEG variability during the neurofeedback therapy.
(4) Developing BCIs for individual use: current methods of NF-training require the presence of an expert to conduct the training session from the installation of scalp electrode to running the programs and maintaining the system. These procedures impose restrictions on the usage of BCIs by patients. More user-friendly, highly automated BCIs should be developed in the future.

## Conclusions

BCIs offer exciting opportunities for enhancing neural functions and developing therapies for neural disabilities, including BCIs that assist subject to regulate their neural function. Attention is a fundamental brain mechanism for selection of relevant and essential information while suppressing irrelevant signals. Disorders of this mechanism result in dysfunctions, such as ADHD. BCIs hold promise to provide effective rehabilitation strategies for individuals with impairments of attention. Several attention-based BCIs have been already developed whereas many challenges still remain. The main challenge is to combine highly technical knowledge needed to build effective BCIs with the expertise from neuroscience and psychology. Merging these multidisciplinary contributions is key to developing clinically relevant BCIs to treat attentional dysfunctions.

## Author contributions

MO, ML: wrote the paper; HS, SP: edited the paper.

### Conflict of interest statement

The authors declare that the research was conducted in the absence of any commercial or financial relationships that could be construed as a potential conflict of interest.
